# Spillover effects of HIV testing policies: changes in HIV testing guidelines and HCV testing practices in drug treatment programs in the United States

**DOI:** 10.1186/s12889-016-3322-4

**Published:** 2016-07-29

**Authors:** Jemima A. Frimpong, Thomas D’Aunno, Stéphane Helleringer, Lisa R. Metsch

**Affiliations:** 1 Department of Health Policy and Management, Mailman School of Public Health, Columbia University, 600 West 168th Street, New York, NY 10032 USA; 2 Robert F. Wagner Graduate School of Public Service, New York University, 295 Lafayette Street, New York, NY 10012 USA; 3 Department of Population, Family and Reproductive Health, Bloomberg School of Public Health, Johns Hopkins University, 615 N. Wolfe Street, Baltimore, MD 21205 USA; 4 Department of Sociomedical Sciences, Mailman School of Public Health, Columbia University, 722 West 168th Street, New York, NY 10032 USA

**Keywords:** HIV testing policies, HIV testing guidelines, HIV pretest counseling, HCV testing, Drug treatment, Programs, Methadone, Buprenorphine, Centers for disease control and prevention

## Abstract

**Background:**

To examine the extent to which state adoption of the Centers for Disease Control and Prevention (CDC) 2006 revisions to adult and adolescent HIV testing guidelines is associated with availability of other important prevention and medical services. We hypothesized that in states where the pretest counseling requirement for HIV testing was dropped from state legislation, substance use disorder treatment programs would have higher availability of HCV testing services than in states that had maintained this requirement.

**Methods:**

We analyzed a nationally representative sample of 383 opioid treatment programs from the 2005 and 2011 National Drug Abuse Treatment System Survey (NDATSS). Data were collected from program directors and clinical supervisors through telephone surveys. Multivariate logistic regression models were used to measure associations between state adoption of CDC recommended guidelines for HIV pretest counseling and availability of HCV testing services.

**Results:**

The effects of HIV testing legislative changes on HCV testing practices varied by type of opioid treatment program. In states that had removed the requirement for HIV pretest counseling, buprenorphine-only programs were more likely to offer HCV testing to their patients. The positive spillover effect of HIV pretest counseling policies, however, did not extend to methadone programs and did not translate into increased availability of on-site HCV testing in either program type.

**Conclusions:**

Our findings highlight potential positive spillover effects of HIV testing policies on HCV testing practices. They also suggest that maximizing the benefits of HIV policies may require other initiatives, including resources and programmatic efforts that support systematic integration with other services and effective implementation.

## Background

To increase HIV testing, promote earlier detection of HIV infection, counsel and link infected persons to HIV clinical and prevention services, the Centers for Disease Control and Prevention (CDC) published revised recommendations for HIV testing in health care settings for adults and adolescents in 2006 [1]. These recommendations stated that general consent for medical care is sufficient for HIV testing, thus eliminating the need for a separate written consent prior to HIV testing. The CDC also recommended eliminating mandatory pretest counseling about risk behaviors and risk reduction. Several studies have indeed found that pretest counseling was time-consuming and placed a high burden on staff members, while having limited impact on subsequent risk of infection with HIV or other sexually transmitted infections (STIs) [2–4]. A recent randomized controlled trial conducted in drug treatment centers found that there were no significant differences in the acquisition STIs and transmission of HIV between groups that received pretest counseling and those that did not [5, 6].

While CDC recommendations may influence state laws, the authority to set or modify HIV testing policies is under the jurisdiction of states. States must decide to amend laws to conform or at a minimum to not conflict with CDC recommendations [7]. By 2011, forty-six states and jurisdictions (including Washington D.C.) had adopted legislation that was compatible with the 2006 CDC guidelines for consent and counseling [8]. These legislative changes appear to have improved the availability and range of HIV services offered at health facilities, as well as the number of patients learning their HIV status [5, 6, 9–13].

The effects of HIV-related legislative changes on the availability of other medical services, on the other hand, have seldom been investigated, even though such effects may be present. The elimination of HIV pretest counseling, for example, may considerably reduce the need for human and financial resources dedicated to HIV testing. These resources could then be redirected towards offering new preventive services or to improving the quality of existing services [4, 14]. Similarly, investments in equipment or human resources required to respond to the increased volume of HIV testing may also present a prime opportunity for providing additional prevention, testing or treatment services more widely and efficiently. Lastly, increased emphasis on HIV testing may influence attitudinal changes among clinical and managerial staff. This may lead to increased support for preventive services more generally.

Services addressing infection with the Hepatitis-C Virus (HCV) may be particularly affected by changes in HIV-related legislation. This is so because both viruses partially share routes of transmission and co-infection is common [15, 16]. HIV and HCV co-infection is common in the U.S., with nearly 25 % of HIV-infected individuals also infected with HCV [17, 18]. HIV and HCV testing also share similar testing modalities. As for HIV testing protocols based on enzyme immunoassays (EIA), common testing protocols for HCV require phlebotomy and follow a two-step process: first, blood samples are tested for the presence of HCV antibodies, then reactive results are confirmed through ribonucleic acid (RNA) testing. A rapid test for HCV antibodies also exists but has been approved by the US Food and Drug Administration (FDA) significantly later than rapid HIV tests. Unlike HIV testing, the CDC has not published specific consent or pretest counseling guidelines for HCV testing. Legislative changes designed to increase the availability and uptake of HIV-related services may thus potentially lead to changes in the availability of HCV-related services.

Such “spillover effects” are important because they may optimize the efficiency and effectiveness of service delivery, through integration of care, and the availability of comprehensive services. These models of care have the potential to increase access, quality, and health outcomes [19, 20]. Spillover effects also have implications that may affect assessments of the cost-effectiveness of HIV-related policies [21].

In this paper, we examined whether the adoption of state legislation compatible with the 2006 CDC guidelines on HIV testing was associated with subsequent improvements in the availability of HCV testing services. We focus on the elimination of the requirement for HIV pretest counseling in opioid treatment programs (OTPs). OTPs are physical facilities with resources dedicated specifically to treating opiate dependence through methadone or buprenorphine (excluding primary care or physician offices). OTPs treat approximately 26% of all individuals enrolled in substance use disorder (SUD) treatment programs the Unites States [22]. The prevalence of HIV and HCV is high among patients of OTPs, particularly persons who inject drugs [23–25]. In addition, whereas persons who use or inject drugs are less likely to regularly seek services in primary healthcare settings [26, 27], they frequently attend SUD treatment facilities. OTPs thus constitute an important setting for increasing the diagnosis of HIV, HCV and other infectious diseases among persons with SUD.

We hypothesized that in states where the pretest counseling requirement for HIV testing was dropped from state legislation, OTPs would have higher availability of HCV testing services than in states that had maintained this requirement. Indeed, simplifying HIV testing protocols may help OTPs invest time and resources in the provision of HCV testing services to their clients. An increasing number of OTPs are buprenorphine-only programs: they represented 14 % of all OTPs in 2005 vs. 27 % in 2011 [28]. Buprenorphine-only programs are often smaller programs, with fewer human resources and connections to hospitals or laboratories. They are also the least likely type of OTPs to provide HIV testing [9] and/or HCV testing [29] to their patients. Programs that offer methadone, on the other hand, more frequently offer a broad range of medical and preventive services to their patients. The association between changes in HIV testing policies and the supply of HCV testing services may thus vary between buprenorphine-only and methadone OTPs.

## Methods

### Data sources

We analyzed data from the National Drug Abuse Treatment System Survey (NDATSS). The NDATSS is a nationally-representative survey, which examines the organizational structures and operating characteristics of the nation’s outpatient SUD treatment programs [30]. Because the Substance Abuse and Mental Health Services Administration (SAMHSA) certifies OTPs, we obtained lists that precisely identify the entire US population of approved OTPs as the sampling frame. The sample is nationally representative, although each wave may not include treatment OTPs from all states due to sampling variation. In addition, the SAMHSA list only includes OTPs, i.e., facilities that are dedicated to the treatment of opioid dependence. Our sampling frame thus does not include primary healthcare facilities that dispense buprenorphine. Primary health care facilities are not registered by SAMHSA. Rather, the requirement is that health care providers undergo a short training course and obtain a US Drug Enforcement Administration (DEA) registration number in order to prescribe buprenorphine. OTPs that prescribe buprenorphine (because they have a provider with a DEA registration number) are included in our sample. The Columbia University Institutional Review Board reviewed and approved the study. Informed consent to participate in the study was obtained orally prior to administering the surveys. During each wave of the NDATSS, the administrative director and clinical supervisor at each of the participating programs were asked to complete a telephone survey on treatment practices and program characteristics. Only aggregate anonymous data on patient care and services were collected from programs. The NDATSS team never reviewed individual patient records. The reliability and validity of the NDATSS, as well as detailed description of the sampling frame and sample, are available from other sources [9, 29, 31]. We used data from all OTPs surveyed in the 2005 (*n* = 187) and 2011 (*n* = 196) NDATSS.

### Dependent variables

HCV testing practices in OTPs were ascertained from NDATSS questions that asked whether OTPs offered any option for HCV testing to their patients, and if so, whether such services were provided on-site or off-site. We constructed two dependent variables from answers to these questions. First, we created a binary variable (0–1) set to 1 if the program offered any HCV testing option to their patients (either on-site or off-site) and 0 otherwise. Given the greater coordination and investment that may be required to offer on-site HCV testing compared to off-site testing, a second binary variable examined the availability of on-site HCV testing services, among OTPs that offered any HCV testing option to their patients. OTPs providing on-site HCV testing were coded 1, and those providing only off-site services were coded 0.

### Classification of states’ legislations

A variable describing state-level HIV-related legislation was constructed based on three previous studies of states’ HIV testing laws. Wolf et al. [32] and Mahajan et al. [7] examined state HIV laws in 2004 and 2008, respectively, whereas Neff and Goldschmidt [8] examined state law compliance with CDC recommendations in 2011. With these data, we generated a dichotomous variable to reflect the compatibility of state-level legislation with CDC guidelines for the elimination of HIV pretest counseling. States were coded 1 if their legislation allowed eliminating the requirement for pretest counseling (adopted CDC guidelines), and 0 (did not adopt CDC guidelines). The resulting classification of states in 2005 and 2011 appears in Table 1. In 2005, some states had already adopted legislation that did not require pretest counseling.

**Table 1:** Adoption of CDC HIV pretest counseling guidelines in states included in the National Drug Abuse Treatment System Survey (NDATSS)

	2005		2011	
	Eliminated HIV Pretest Counseling	N	Eliminated HIV Pretest Counseling	N
Alabama	N	6	Y	4
Alaska	N	--	Y	1
Arizona	N	2	Y	7
Arkansas	N	1	Y	1
California	N	25	Y	16
Colorado	N	5	Y	3
Connecticut	N	2	Y	3
Delaware	N	1	Y	1
DC	N	3	Y	--
Florida	N	4	Y	5
Georgia	Y	3	Y	5
Hawaii	N	--	Y	1
Illinois	Y	10	Y	14
Indiana	N	2	Y	1
Iowa	N	1	Y	1
Kansas	N	2	Y	3
Kentucky	N	2	Y	2
Louisiana	N	2	Y	3
Maine	N	5	Y	1
Maryland	Y	8	Y	9
Massachusetts	N	7	Y	3
Michigan	N	4	Y	4
Minnesota	N	1	Y	3
Missouri	N	1	Y	4
Montana	N	1	Y	1
New Jersey	N	7	Y	6
New Mexico	Y	2	Y	2
New York	N	28	Y	38
North Carolina	N	1	Y	3
Ohio	N	9	Y	13
Oklahoma	N	1	N	--
Oregon	N	5	Y	2
Pennsylvania	N	18	N	13
Rhode Island	N	--	N	1
South Carolina	N	1	Y	1
Tennessee	N	1	N	--
Texas	N	7	Y	9
Utah	N	2	Y	2
Vermont	N	1	Y	1
Virginia	N	2	Y	4
Washington	N	2	Y	4
West Virginia	N	1	Y	1
Wisconsin	N	1	N	--

Note: The table shows elimination of HIV pretest counseling in states included in the NDATSS. Y = State legislation eliminated pretest counseling; N = state retained legislation that did not permit eliminating pretest counseling

### Control variables

OTPs that treat a large proportion of patients with characteristics indicating high-risk for HIV or HCV may have greater likelihood of offering preventive services. Patient factors accounted for in the models therefore included prevalence of injection drug users (IDU) among patients in each program and total number of OTP patients in the past year and race/ethnicity [33]. Organizational characteristics and resources may also influence treatment practices [29, 34, 35]. We adjusted for several characteristics of OTPs including: sources of revenue (federal government, private insurance), staff to patient ratio, Commission on Accreditation of Rehabilitation Facilities (CARF) accreditation, ownership (private for profit, private not for profit, public) and hospital affiliation. We controlled for program type (methadone-only, buprenorphine-only, both methadone and buprenorphine), which has been associated with availability of HIV and HCV testing services [9, 29]. Finally, we also controlled for time trends in the adoption of HCV testing in OTPs by including a binary variable identifying observations from the 2011 survey (1 = 2011, 0 = 2005).

### Data analysis

We first reviewed state policies about HIV pretest counseling in 2005 and 2011 and assessed their compatibility with the 2006 CDC guidelines. We then described differences in OTP characteristics between programs offering any HCV testing services (either on-site or off-site) to their patients vs. OTPs in which no HCV testing services were offered. We used the Pearson χ^2^ test for categorical variables and the *t* test for continuous variables, and we computed unadjusted odds ratios. Second, we estimated multivariate logistic regression models to examine associations between state-level HIV-related legislation and availability of HCV testing services in OTPs, controlling for program and patient characteristics [36]. Third, we used multivariate logistic regressions to test whether, among OTPs that did offer HCV testing services, state-level HIV-related legislation was associated with increased adoption of on-site HCV testing among OTPs that offered HCV testing option to their patients. Finally, we tested whether the association between state-level HIV-related legislation and HCV testing services varied by type of drug treatment (programs that offer buprenorphine only vs. programs that offer methadone, either methadone only or methadone and buprenorphine) provided in OTPs. We did so by including an interaction term in our logistic regression models.

In all models, we addressed issues of unobserved heterogeneity (i.e., confounding variables that may affect our estimates of the association between state legislation and the availability of HCV testing at OTPs) by including state fixed-effects [37]. This strategy permits controlling for all time-invariant characteristics of states that may be associated with both the likelihood of adopting legislation that enables changing the pretest counseling requirement for HIV testing and the availability of HCV testing in OTPs. We adjusted all standard errors for clustering of observations within state. All analyses were carried out using the STATA 12.0 (StataCorp LP, College Station, TX).

## Results

Only 4 states included in NDATSS had already adopted HIV-related legislation that eliminated the requirement for pretest counseling at the time of the 2005 NDATSS wave. These included Georgia, Illinois, Maryland, and New Mexico. On the other hand, most states had adopted such legislation by 2011: only six states or jurisdictions (Washington D.C., Oklahoma, Pennsylvania, Rhode Island, Tennessee, and Wisconsin) in the 2011 NDATSS had retained legislation that did not permit dropping the pretest counseling risk (Table 1).

Table 2 describes differences in the characteristics between OTPs in which HCV testing options were offered to patients and OTPs where no HCV testing options were offered. In bivariate analyses, offer of HCV testing services in OTPs was associated with a lower proportion of African-Americans among patients, a more favorable staff-to-patient ratio, CARF accreditation and method of treatment. OTPs, which offered both methadone and buprenorphine to their patients, were more likely to also offer HCV testing services than other OTPs. OTPs located in states where HIV-related legislation enabled dropping the pretest counseling requirement had higher availability of HCV testing services (87.3 % vs. 75.3 %, *p* < 0.01). In multivariate analyses with controls for organizational characteristics and state-level fixed effects, however, there were no significant differences in HCV testing availability between states whose legislation permitted dropping the HIV pretest counseling requirement and other states (aOR = 0.87, 95 % CI = 0.19, 4.07).

**Table 2:** Regression analyses of the association between adoption of CDC HIV pre-test counseling guidelines and availability of HCV testing (HCV testing vs. No HCV testing)

	No HCV testing options offered^a^	HCV testing offered	*P*-value	Unadjusted Odds Ratio (95 % CI)^b^	Adjusted Odds Ratio (95 % CI)^b^
State-level legislation enables eliminating HIV pretest counseling requirement			<0.01		
No	44 (24.7)	134 (75.3)		1	1
Yes	26 (12.7)	179 (87.3)		2.26 (1.32, 3.86)	0.87 (0.19, 4.07)
Prevalence of injection drug users			0.07		
< 25 %	33 (24.8)	100 (75.2)		1	1
25-74 %	24 (14.9)	137 (85.1)		1.88 (1.05, 3.39)	2.21 (0.97, 5.04)
≥ 75 %	13 (15.5)	71 (84.5)		1.80 (0.89, 3.67)	1.44 (0.51, 4.11)
African-American patients			0.05		
< 10 %	24 (13.9)	148 (86.1)		1	1
≥ 10 %	46 (21.8)	165 (78.2)		0.58 (0.34, 0.99)	0.85 (0.37, 1.94)
Hispanic patients			0.17		
< 10 %	41 (20.9)	155 (79.1)		1	1
≥ 10 %	29 (15.5)	158 (84.5)		1.44 (0.85, 2.44)	1.27 (0.54, 2.97)
Revenue from federal government			0.85		
None	50 (18.5)	220 (81.5)		1	1
≥ 1 %	20 (17.7)	93 (82.3)		1.06 (0.60, 1.87)	1.15 (0.53, 2.49)
Revenue from private insurance			0.18		
None	35 (16.0)	184 (84.0)		1	1
≥ 1 %	35 (21.3)	129 (78.7)		0.70 (0.42, 1.18)	1.11 (0.51, 2.43)
Human resources					
Log Staff-to-patient ratio, mean (SD)	-3.56 (0.76)	-3.31 (0.72)	0.01	1.53 (1.09, 2.16)	1.30 (0.82, 2.06)
CARF accreditation			0.01		
No	45 (23.2)	149 (76.8)		1	1
Yes	25 (13.2)	164 (86.8		1.98 (1.16, 3.39)	2.23 (0.98, 5.08)
Ownership			0.78		
Private not-for-profit	36 (19.6)	148 (80.4)		1	1
Private for profit	24 (16.6)	121 (83.4)		1.22 (0.69, 2.17)	0.91 (0.40, 2.08)
Public	10 (18.5)	44 (81.5)		1.07 (0.49, 2.33)	0.97 (0.33, 2.80)
Hospital affiliation			0.09		
No	64 (19.7)	261 (80.3)		1	1
Yes	6 (10.3)	52 (89.7)		2.12 (0.87, 5.17)	3.39 (1.13, 10.2)
Methods of treatment			<0.01		
Methadone only	45 (20.7)	172 (79.3)		1	1
Buprenorphine only	22 (23.2)	73 (76.8)		0.87 (0.49, 1.55)	1.55 (0.62, 3.88)
Methadone + Buprenorphine	3 (4.2)	68 (95.8)		5.93 (1.78, 19.8)	6.63 (1.61, 27.4)
Time			<0.01		
2005	50 (26.7)	137 (73.3)		1	1
2011	20 (10.2)	176 (89.8)		3.21 (1.82, 5.65)	3.20 (0.87, 11.7)
N	70 (18.3)	313 (81.7)		383	292^c^

Notes: ^a^percentages in parentheses are row percentages; ^b^adjusted odds ratios are obtained from a logistic regression in which all variables in the table are included as independent variables. Standard errors are adjusted for clustering of observations by state. ^c^Only states in which laws changed between 2005 & 2011 are included in model with state-level fixed effects. *p*-value derived from a Wald test showed that at least one of the state dummies included in the model is significant at *p* < 0.05

**Fig. 1:**
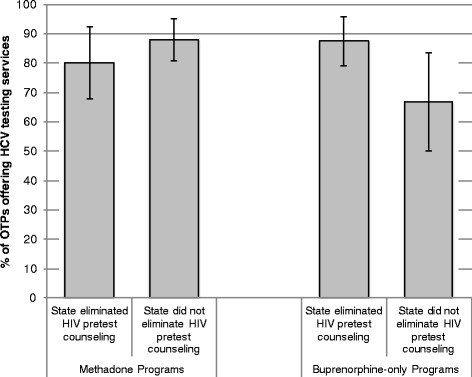
Estimates of the Association between State-level HIV Pre-Test Counseling Policies and Availability of Hepatitis C Testing Services in Opioid Treatment Programs, by Type of Drug Treatment Program. Notes: the figures presented here are predicted probabilities obtained from logistic regressions with state fixed-effects. The analytical sample in these models includes all OTPs in states where the HIV-relation legislation changed between 2005 and 2011. Error bars represent 95% confidence intervals. An interaction term between type of treatment program and adoption statelevel HIV-related legislation was significant at *p*<0.05 level. This model includes controls for other characteristics of OTPs listed in table 2. Standard errors are adjusted for clustering of observations within state

The association between state-level HIV-related legislation and the availability of HCV testing services differed significantly by type of treatment program (Fig. 1, Wald test of interaction *p*-value <0.05). In programs offering methadone-only or methadone and buprenorphine to their patients, 80 % of OTPs located in states that had dropped the requirement for HIV pretest counseling offered HCV testing services vs. 87 % in states that still required pretest counseling. In OTPs that offered only buprenorphine, on the other hand, 88 % of OTPs in states that had dropped the pretest counseling requirement offered HCV testing services vs. only 68 % in states that had maintained this requirement (*p* < 0.05).

In bivariate analyses, among OTPs that offered HCV testing to their patients, the type of HCV testing services offered (on-site vs. off-site referral) was associated with the prevalence of IDUs among patients, funding from the federal government, more favorable staff-to-patient ratios, OTP ownership, hospital affiliation and method of treatment (Table 3). OTPs located in states that had dropped the HIV pretest counseling requirement were less likely to offer on-site HCV testing than OTPs in states that had maintained this requirement (40.8 % vs. 68.7 %, *p* < 0.01). However, in multivariate analyses, there were no significant differences in the likelihood of offering on-site testing between these two types of states (aOR = 1.65, 95 % CI = 0.32, 8.46).

**Table 3:** Regression analyses of the association between adoption of CDC HIV pre-test counseling guidelines and offer of on-site vs. Off-site HCV testing

	Off-site HCV testing^a^	On-site HCV testing	*P*-value	Unadjusted Odds Ratio (95 % CI)^b^	Adjusted Odds Ratio (95 % CI)^b^
State-level legislation enables eliminating HIV pretest counseling requirement			<0.01		
No	42 (31.3)	92 (68.7)		1	1
Yes	106 (59.2)	73 (40.8)		0.31 (0.20, 0.50)	1.65 (0.32, 8.46)
Prevalence of injection drug users			0.06		
< 25 %	57 (57.0)	43 (43.0)		1	1
25-74 %	57 (41.6)	80 (58.4)		1.86 (1.10, 3.14)	2.68 (1.11, 6.47)
≥ 75 %	33 (46.5)	38 (53.5)		1.53 (0.83, 2.82)	0.69 (0.24, 2.01)
African-American patients			0.49		
< 10 %	73 (49.3)	75 (50.7)		1	1
≥ 10 %	75 (45.4)	90 (54.6)		1.17 (0.75, 1.82)	0.97 (0.41, 2.30)
Hispanic patients			0.70		
< 10 %	75 (48.4)	80 (51.6)		1	1
≥ 10 %	73 (46.2)	85 (53.8)		1.09 (0.70, 1.70)	1.26 (0.50, 3.16)
Revenue from federal government			0.01		
None	114 (51.8)	106 (48.2)		1	1
≥ 1 %	33 (36.6)	59 (63.4)		1.87 (1.13, 3.07)	2.47 (1.04, 5.88)
Revenue from private insurance			0.11		
None	80 (43.5)	104 (56.5)		1	1
≥ 1 %	48 (52.7)	61 (47.3)		0.69 (0.44, 1.08)	1.33 (0.59, 2.98)
Human resources					
Log Staff-to-patient ratio, mean (SD)	-3.40 (0.78)	-3.24 (0.66)	0.05	1.36 (0.99, 1.88)	1.80 (1.10, 2.93)
CARF accreditation			0.21		
No	76 (51.0)	73 (49.0)		1	1
Yes	72 (43.9)	97 (56.1)		1.33 (0.85, 2.08)	1.37 (0.60, 3.12)
Ownership			<0.01		
Private not-for-profit	77 (52.0)	71 (48.0)		1	1
Private for profit	61 (50.4)	60 (49.6)		1.07 (0.66, 1.73)	1.52 (0.66, 3.46)
Public	10 (22.7)	34 (77.3)		3.69 (1.70, 8.02)	3.36 (0.95, 11.9)
Hospital affiliation			<0.01		
No	134 (51.3)	127 (48.6)		1	1
Yes	14 (26.9)	38 (73.1)		2.86 (1.48, 5.54)	6.22 (2.04, 18.9)
Methods of treatment			<0.01		
Methadone only	69 (40.1)	103 (59.9)		1	1
Buprenorphine only	55 (75.3)	18 (24.7)		0.22 (0.12, 0.41)	0.17 (0.06, 0.49)
Methadone + Buprenorphine	24 (35.3)	44 (64.7)		1.23 (0.68, 2.20)	2.95 (1.19, 7.31)
Time			<0.01		
2005	38 (27.7)	99 (72.3)		1	1
2011	110 (62.5)	66 (37.5)		0.23 (0.14, 0.37)	0.10 (0.02, 0.45)
N	148 (47.3)	165 (52.7)		313	282^c^

Notes: ^a^percentages in parentheses are column percentages; ^b^adjusted odds ratios are obtained from a logistic regression in which all variables in the table are included as independent variables. Standard errors are adjusted for clustering of observations by state. ^c^Only states in which laws changed between 2005 & 2011 are included in model with state-level fixed effects. *p*-value derived from a Wald test showed that at least one of the state dummies included in the model is significant at *p* < 0.05

**Fig. 2:**
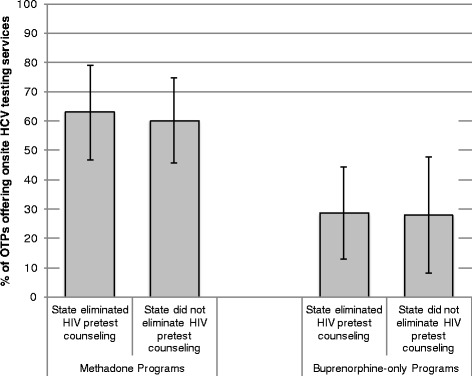
Estimates of the Association between State-level HIV Pre-Test Counseling Policies and Availability of Hepatitis C Testing Services On-site in Opioid Treatment Programs, by Type of Drug Treatment Program. Notes: the figures presented here refer to OTPs, which offer at least some kind of HCV testing services (i.e., on-site or offsite). The figures reported are predicted probabilities obtained from logistic regressions with state fixed-effects. The analytical sample in these models includes all OTPs in states where the HIV-relation legislation changed between 2005 and 2011. Error bars represent 95 % confidence intervals. In this model, there was no significant interaction between type of treatment program and adoption of legislation consistent with CDC guidelines. Standard errors are adjusted for clustering of observations within state

Among OTPs that offered HCV testing services to their patients, there was no interaction between HIV-related legislation and the type of treatment program (Fig. 2) in determining whether HCV testing was offered on-site or off-site. Between 60-62 % of OTPs offering methadone-only or methadone and buprenorphine to their patients had on-site HCV testing vs. 28 % of OTPs offering buprenorphine only to their patients.

## Discussion

In this paper, we examined the potential spillover effects of state-level HIV testing policies on the availability of HCV testing in opioid treatment programs. We hypothesized that in states where the pretest counseling requirement for HIV testing was dropped from state legislation, OTPs would have higher availability of HCV testing services than in states that had maintained this requirement. Indeed, simplifying the procedures for HIV testing may help OTPs mobilize financial and/or human resources for the provision of other medical services, such as HCV testing.

Using data from two recent rounds of the NDTASS, we found that this was not uniformly the case. In OTPs that offered only buprenorphine treatment to their patients for substance abuse treatment, legislative changes that permitted dropping the pretest counseling requirement for HIV testing were associated with significantly higher availability of HCV testing. On the other hand, in OTPs that offered methadone to their patients for substance abuse treatment, there were no differences in HCV testing availability between states with and without a legal requirement for HIV-related pretest counseling.

There are several reasons why the elimination of pretest counseling for HIV testing may affect HCV testing in OTPs that offer only buprenorphine. First, this may be related to the type of patient population served by buprenorphine-only programs [34, 38, 39]. Programs that adopt buprenorphine are more reliant on revenue from private insurance and patient self-pay [40]. They are also less likely to treat persons who inject drugs as a method of substance use [28, 41] and they often treat patients that use other opiates instead of heroin [41]. Buprenorphine-only programs may therefore be less likely to consider their patient population as at-risk for HIV or HCV and to invest in the resources required to provide testing services. The elimination of pretest counseling may have reduced the investment required for offering HIV testing (i.e., time required to provide pretest counseling and complete testing) and may have indirectly influenced the offer of other preventive services, including HCV testing.

Second, we suggest that the removal of the requirement to provide pretest counseling for HIV may have reduced concerns of stigma among the patient population and providers of buprenorphine-only programs. Patients of these programs may indeed be more likely to associate pretest counseling messages with stigmatized behaviors such as injecting drug use, thus reducing their demand for testing services [42, 43]. Streamlining the HIV testing process through the elimination of the pretest counseling requirement may therefore increase the acceptability of testing for HIV and HCV among patients and providers in buprenorphine-only programs. It may help these programs adopt HCV testing as standard medical practice [44].

Finally, there may be aspects of the operating environment of buprenorphine-only programs that make them more responsive to legislative changes than methadone programs. Whereas methadone maintenance programs must be certified by SAMHSA, licensed at the state level and provide directly observed therapy (in some case patients may be eligible for take-home methadone), buprenorphine-only programs solely need to have on staff a health care provider who has obtained DEA approval to prescribe controlled substances. The less structured operational requirements of buprenorphine-only programs may thus make it easier for these programs to invest resources in HCV testing following the adoption of legislative changes. This may account for the observed differences in the association between the removal of the HIV pre-test counseling requirement and HCV testing practices across different types of OTPs.

Our results also indicated that HIV-related legislation did not translate into an increase in the offer of on-site testing among OTPs that offered HCV testing. This is likely sub-optimal since on-site services are significantly more effective at improving uptake of testing among patients of drug treatment programs than off-site services [6, 45]. Changes in HIV-related legislation may not have lead to the adoption of on-site HCV testing for reasons related to the standard HCV testing algorithm. Indeed, until recently HCV anti-body testing was based on collection of blood specimen obtained by venipuncture [46, 47]. Programs that do not have the required human or financial resources to conduct on-site testing (e.g., buprenorphine-only programs) may instead offer testing services through referral sources. However, the introduction and adoption of rapid HCV testing may improve the availability of on-site HCV testing. Rapid HIV testing is often preferred to conventional testing, given the ease of testing and immediacy of results [48, 49]. It is also associated with increased uptake of testing [50–52]. Thus, an increase in on-site HCV testing may be facilitated by the adoption of rapid HCV testing [53]. Interventions that effectively integrate rapid HIV and rapid HCV testing and treatment services are needed [15, 54].

Several other factors may further improve the adoption of HCV testing in OTPs. This includes the 2012 CDC recommendation to expand HCV testing to include persons born between 1945 and 1965 [55], or the advent of highly effective and efficient antiretroviral treatments for HCV [56]. Expansion of testing for HCV is important because similar to HIV, diagnosis and awareness of one’s infection status sets into motion a continuum of care, which in the case of HCV can lead to a cure [57].

Our results improve on previous evaluations of the effects of HIV-related legislation [6, 9, 10, 58, 59] by investigating the “spillover effects” of HIV-related legislation on non-HIV services. Such effects are important to take into account when evaluating the cost-effectiveness of legislative changes. Indeed, improvements in the availability of HCV testing services following modifications of the pretest counseling requirement for HIV testing could benefit patients in multiple ways. They may lead to an earlier detection, and thus better clinical management, of HIV/HCV co-infection. They may lead to reductions in incidence of HIV and/or HCV if patients learning their HIV and/or HCV serostatus modify their risk behaviors and adopt protective measures. The spillover effects of HIV-related legislations may also extend beyond the availability of HCV testing services. For example, dropping the requirement for HIV pretest counseling may allow health workers in SUD treatment programs to spend more time on substance use counseling. This may in turn lead to increasing SUD treatment completion rates and/or improving patient outcomes (e.g., sobriety at treatment completion, fewer relapses). It may also improve the availability of a broad range of other medical services such as vaccination against hepatitis B, STI testing and similar diseases. The impact of changes in HIV-related legislation on epidemiological trends and estimates of healthy life expectancy may thus be larger than previously thought [60, 61].

While HIV testing policies may have a spillover effect on HCV testing practices, increasing the availability of HCV testing will require a multi-pronged approach. Consent and counseling guidelines focused on HCV may inform state policies on the issue, but state adoption of existing guidelines and their direct effects on HCV testing practices has not been examined [16, 55]. Future studies should therefore systematically assess variations in HCV testing polices across states and their impact on OTPs’ testing practices. Additionally, adequate resources must be devoted to the prevention, control and surveillance of HCV [62]. Unfortunately, the U.S. Department of Health and Human Services report on “Action Plan to Prevent, Care and Treat Viral Hepatitis”, which was released in 2011 and updated in 2014, did not include a provision to increase funding for viral hepatitis [63]. The Affordable Care Act (ACA) expansion of coverage for treatment of SUDs and hepatitis prevention, diagnosis, care, and treatment however present a promising pathway to improving access and patient outcomes [64]. Additionally, as newer more effective medications for HCV become available, it would be important to examine whether and how lessons learned about the HIV care continuum could be applied to the HCV care continuum to improve case identification and treatment outcomes [65].

This study has several limitations. First, the NDATSS had limited questions on patients of OTPs. We therefore could not measure the relationship between HIV pretest counseling policies and uptake of HCV testing services among patients. Second, we also could not determine the influence of length of time since elimination of pretest counseling on HCV testing practices. Specifically, we do not know how long after a State changed its legislation, the OTPs in that State changed their HIV testing practices, including removal of the pre-test counseling requirement. This is important because an OTP with more experience implementing the guidelines, e.g., a program operating under elimination of pretest counseling for a year, may have better knowledge about the influence of the policy on operating procedures, and may therefore be more likely to offer HCV testing services. On the other hand, a program that has newly eliminated pretest counseling may be less likely to offer HCV testing services. Third, we performed separate analyses to assess the associations between State legislation on HIV testing and a) HCV testing (either on-site or off-site), as well as b) on-site HCV testing in OTPs. This was necessary because of limited sample size. Future studies should rely on larger samples, and should employ other methodological approaches (multinomial regression) to further extend our findings. Lastly, the NDATSS data do not permit assessing whether the relation between HIV testing policies and HCV testing practices is causal. Even though we included state-level fixed-effects in our models, these only control for time-invariant characteristics of states that may confound the relationship between legislative changes and HCV testing. They do not account for a) characteristics of states that changed between 2005 and 2011, and b) unobserved characteristics of OTPs. Addressing this issue requires panel data on OTPs and their practices, i.e., a sample of OTPs that are repeatedly surveyed over time. Unfortunately, only a small number of OTPs in the NDATSS were interviewed both in 2005 and 2011, thus precluding such robust causal analyses. In addition, our data does not permit determining whether OTPs adopted HCV testing before or after the State in which they are located changed its HIV-related legislation. This is so because we only know whether surveyed OTPs offered HCV testing in 2005 or 2011. We do not know when exactly they first adopted HCV testing. As a result, we can only identify associations between HIV-related state laws and HCV testing practices in OTPs.

## Conclusions

Our results highlight some of the implications of HIV policies for the availability of non-HIV services. Buprenorphine-only programs were more likely to offer HCV testing services in states that mandated the elimination of HIV pretest counseling, but this was not the case for methadone programs. Changes in legislation related to HIV pretest counseling also were not associated with the availability of on-site HCV testing. Our findings underscore several areas for future research. There is a need for a better understanding of the mechanisms (i.e., managerial decision-making) or pathways (i.e., translation of polices in service delivery settings) through which HIV pretest counseling policies influence non-HIV services. Additionally, studies should investigate the impact of HIV testing policies on HCV testing services, considering state guidelines for HCV testing. Lastly, the longer-term effect of eliminating HIV pretest counseling on clients and their use of HIV-related services, and whether programs introducing HCV testing after the elimination of HIV pretest counseling provide pre-test counseling for HCV testing should be examined in future studies. These analyses will provide a more in-depth understanding of the role of policy interventions in shaping HCV testing practices, promote the effective integration of HIV and HCV services, and increase the availability of HCV testing in substance abuse treatment programs and other health care settings.

## Abbreviations

ACA, affordable care act; CARF, commission on accreditation of rehabilitation facilities; CDC, centers for disease control and prevention; DEA, drug enforcement administration; EIA, enzyme immunoassays; FDA, food and drug administration; HCV, hepatitis c virus; HIV, human immunodeficiency virus (HIV); IDU, injection drug users; NDATSS, national drug abuse treatment system survey; OTPs, opioid treatment programs; RNA, ribonucleic acid; SAMHSA, substance abuse and mental health services administration; STI, sexually transmitted infections; SUD, substance use disorder
